# Molecular Dynamics Simulation of Polyacrylamide Adsorption on Calcite

**DOI:** 10.3390/molecules28176367

**Published:** 2023-08-31

**Authors:** Keat Yung Hue, Jin Hau Lew, Maung Maung Myo Thant, Omar K. Matar, Paul F. Luckham, Erich A. Müller

**Affiliations:** 1 Department of Chemical Engineering, Imperial College London, London SW7 2AZ, UK; v.hue20@imperial.ac.uk (K.Y.H.); s.lew20@imperial.ac.uk (J.H.L.); o.matar@imperial.ac.uk (O.K.M.); p.luckham01@imperial.ac.uk (P.F.L.); 2 PETRONAS Research Sdn. Bhd., Lot 3288 & 3289, Off Jalan Ayer Itam, Kawasan Institusi Bangi, Kajang 43000, Selangor, Malaysia; maungmyothant@petronas.com.my

**Keywords:** adsorption, molecular simulation, AFM, surfaces, polymers

## Abstract

In poorly consolidated carbonate rock reservoirs, solids production risk, which can lead to increased environmental waste, can be mitigated by injecting formation-strengthening chemicals. Classical atomistic molecular dynamics (MD) simulation is employed to model the interaction of polyacrylamide-based polymer additives with a calcite structure, which is the main component of carbonate formations. Amongst the possible calcite crystal planes employed as surrogates of reservoir rocks, the (1 0 4) plane is shown to be the most suitable surrogate for assessing the interactions with chemicals due to its stability and more realistic representation of carbonate structure. The molecular conformation and binding energies of pure polyacrylamide (PAM), hydrolysed polyacrylamide in neutral form (HPAM), hydrolysed polyacrylamide with 33% charge density (HPAM 33%) and sulfonated polyacrylamide with 33% charge density (SPAM 33%) are assessed to determine the adsorption characteristics onto calcite surfaces. An adsorption-free energy analysis, using an enhanced umbrella sampling method, is applied to evaluate the chemical adsorption performance. The interaction energy analysis shows that the polyacrylamide-based polymers display favourable interactions with the calcite structure. This is attributed to the electrostatic attraction between the amide and carboxyl functional groups with the calcite. Simulations confirm that HPAM33% has a lower free energy than other polymers, presumably due to the presence of the acrylate monomer in ionised form. The superior chemical adsorption performance of HPAM33% agrees with Atomic Force Microscopy experiments reported herein.

## 1. Introduction

In reservoirs characterised by poorly consolidated carbonate rocks, the probability of producing undesired fine particles is high, which poses the risk of increasing environmental waste and damaging surface equipment [1]. To overcome the problem, a formation-strengthening chemical is typically applied to reduce the solids production risk. By injecting the chemical into the reservoirs, the formation compressive strength will be improved by bonding formation grains, thereby enhancing the intergranular forces. However, screening chemicals can be experimentally tedious and time-consuming. 

The formation-strengthening chemicals can be screened in terms of the chemicals’ adsorption performance on the solid surface, and in this sense, molecular dynamics (MD) simulation is a useful in silico tool to complement experimental studies in this area. It provides information on fluid-solid interactions from an atomistic level [2]. The interactions are assessed through equilibrium adsorption simulation [3,4,5,6] or free energy analysis with an enhanced sampling method [7,8,9,10,11].

Among the plausible chemical candidates, polyacrylamide-based (PAM) polymers stand out due to their excellent viscosifying properties. PAM polymers have been utilised as flocculation agents in wastewater treatment and gelling agents in energy applications [12,13]. The polymers can be easily synthesised and hydrolysed with different copolymers to improve the adsorption performance [14]. Some examples include hydrolysed polyacrylamide (HPAM) and sulfonated polyacrylamide (SPAM), which SPAM has a bulkier copolymer of deprotonated 2-acrylamide-tertiary-butyl sulfonic acid and exhibit higher thermal stability as a polymer flooding agent [13,15]. There is a well-established guideline for the evaluation and selection of these water-soluble polymers in different operating conditions [16]. 

The most representative mineral for carbonate rocks is calcite, which has a rhombohedral crystal structure belonging to the space group of *R*$\bar{3}$*c*. It can exist in different surface planes, where the calcite (1 0 4) plane has been experimentally demonstrated to be the most thermodynamically stable structure due to its low surface free energy [3,17,18]. Molecular simulations of adsorption on calcite with different crystal plane models have been reported in the literature. Cooke et al. [19] studied the competitive adsorption between ethanol and water on a (1 0 4) calcite surface. They found that ethanol dominates the adsorption and forms an adsorption layer over the calcite surface, decreasing the ability of calcite to interact with water. Lowry et al. [20] simulated the removal of polar crude oil components from calcite (1 0 4) using surfactants and microemulsions. These components enable the adsorption of non-polar oil components, making the carbonate oil wet. However, the wettability can be altered with the non-ionic surfactant, which assists the oil recovery process. A similar effect of surfactant on oil displacement had been reported by Yuan et al. but using the (0 0 1) calcite model [21], while Liu et al. [22] and Zhong et al. [23] studied the oil desorption process on the mineral surface of silica. 

The performance of polymer adsorption on surfaces has also been reported in the literature, with one example corresponding to PAM adsorption on cellulose nanocrystals in the polymer nanocomposite field [24]. In the mining industry, hydrolysed polyacrylamide (HPAM) polymer is used as a flocculation or coagulation agent to treat wastewater [25]. Quezada et al. [26] conducted molecular dynamics (MD) simulations to investigate HPAM adsorption on silica surfaces in saltwater environments with different pH levels. They rationalised that salt cations act as a bridging agent to enhance the adsorption of HPAM onto silica while increasing pH and charge density induce negative repulsion forces and reduce the adsorption rate. In biotechnology applications, Sparks et al. simulated polyacrylic acid (PAA) adsorption onto calcite surfaces in the water phase at various pH levels [27]. The polymer adopts an extended conformation during adsorption, decreasing the adsorption energy with increasing pH. In energy applications, Ahsani et al. [28] conducted a simulation study and showed that the adsorption of HPAM on calcite can alter the calcite surface to become water-wet and facilitate oil molecules removal, while another study reported that the effectiveness of HPAM is limited in high-salinity environments due to the neutralisation of the HPAM chains [29].

As a background to this work, Lew et al. [30] investigated the adsorption of HPAM with different molecular weights onto calcium carbonate (CaCO_3_). Their results suggested that HPAM adsorbs readily onto CaCO_3_, with the equilibrium amount of HPAM adsorbed onto CaCO_3_ increasing with polymer molecular weight. This finding is similar to that of Rasteiro et al. [31], where a higher equilibrium adsorbed amount could be obtained with PAM of more considerable molecular weight. They explained that lower molecular weight PAM tends to adopt a flatter configuration on the substrate surface, with each section occupying a more significant portion of the surface, effectively hindering subsequent adsorption. A particularly useful experimental technique to study polymer interactions with substrate surfaces is Atomic Force Microscopy (AFM). One relevant experimental study was conducted by Ekanem et al. [32], where the authors discussed the adsorption of high molecular weight HPAM onto a freshly cleaved calcite crystal. Their AFM results showed that HPAM exhibits high surface coverage over the calcite surface with different regions of high, medium, and low HPAM retention. They proposed that the high HPAM coverage is due to the attraction between charged carboxylate group on the polymeric segments of HPAM and the positively charged calcite surface [27,33]. 

To summarise, the literature shows that while there are reports of simulations on either calcite and/or polyacrylamide adsorption available, they are not immediately transferrable to the solids production control problem and screening of potential polymer candidates. Moreover, the role of the calcite crystal plane model and its inherent flexibility have not been consistently considered [19,20,21,34]. This manuscript presents an integrated experimental and simulation study to identify the key features of the adsorption of polyacrylamide-based polymers onto calcite surfaces. 

## 2. Results and Discussion

### 2.1. Crystal Plane Evaluation and Solid-Fluid Interactions

**Figure 1:**
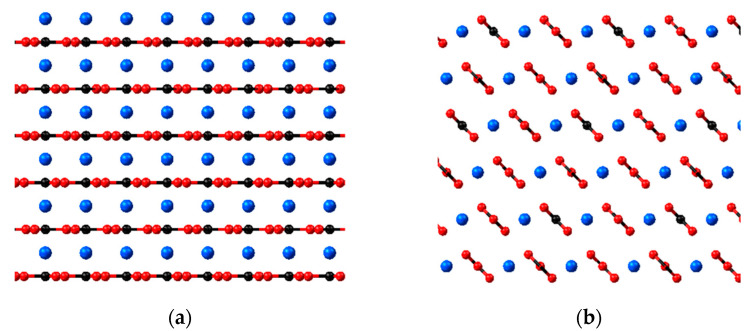

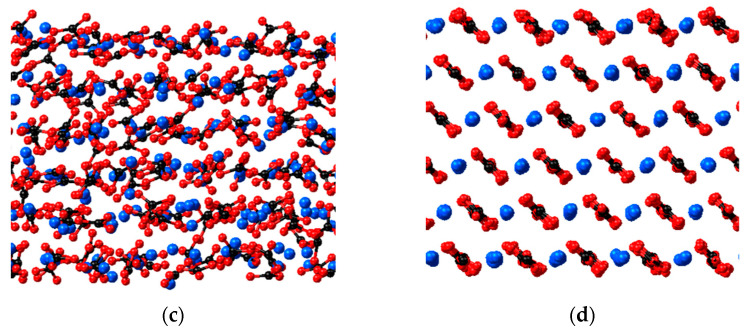
Calcite structures with different crystal planes. (**a**) initial calcite (0 0 1), (**b**) initial calcite (1 0 4), (**c**) calcite (0 0 1) after equilibration and (**d**) calcite (1 0 4) after equilibration.

The initial configuration of calcite structure (0 0 1) and (1 0 4) are shown in Figure 1a and Figure 1b, respectively. It can be observed that the main difference between the two crystal planes is the orientation of the Ca^2+^ ions and CO_3_^2−^ ions. The Ca^2+^ ions layer alternates with the CO_3_^2−^ ions layer for calcite (0 0 1), while both Ca^2+^ ions and CO_3_^2−^ ions are in the same layer for calcite (1 0 4). In our preliminary study, when both structures are allowed to equilibrate freely, the calcite (0 0 1) vibrates noticeably and looks more unstable compared to the calcite (1 0 4), where the atoms equilibrate in their fixed positions, as depicted in Figure 1c,d. This is due to the orientation nature of the atoms in the (0 0 1) surface plane, where the cations are in the same layer followed by another layer of anions, resulting in a dipolar upper and lower surface. The electrostatic repulsion between the ions with the same charges forces them to push away from each other, but they are locked in the crystal lattice position, making the structure inherently unstable. For the calcite (1 0 4) plane, as the Ca^2+^ and CO_3_^2−^ ions are arranged alternately with each other, each cation is stabilised with the presence of another anion. The potential energy is minimised during the equilibration stage, and they can equilibrate more freely in a stable state. 

**Figure 2:**
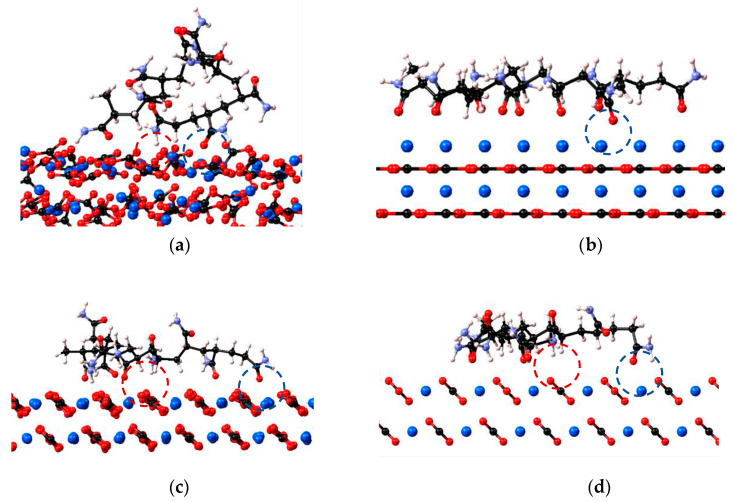
(**a**) PAM with flexible calcite (0 0 1), (**b**) PAM with rigid calcite (0 0 1), (**c**) PAM with flexible calcite (1 0 4) and (**d**) PAM with rigid calcite (1 0 4). PAM atoms colours- light blue atom: N, white atom: H, black atom: C, red atom: O. Red dashed circle indicates the electrostatic interaction between the polymer H atom and calcite O atom. The blue dashed circle highlights the electrostatic interaction between the polymer O atom and calcite Ca atom.

The polymer interaction behaviour with calcite (0 0 1) and calcite (1 0 4) is further tested by placing PAM on the calcite structure in the flexible and rigid state, where the motivation is described in the methods section. The adsorption conformation of polymer PAM to calcite (0 0 1) in the flexible and rigid surfaces are depicted in Figure 2a and Figure 2b, respectively, while the adsorption conformation of polymer PAM unto calcite (1 0 4) in flexible and rigid cases are shown in Figure 2c,d. PAM mainly adopts a flat and extended conformation on the calcite surface, except for the case of PAM with flexible calcite (0 0 1), where the PAM adopts a coiled conformation. Given that these simulations are performed in vacuo, the extended conformations suggest that the polymer can relax on the surface and extend the chain to interact favourably with the calcite surface. On the other hand, as observed in the PAM with flexible calcite (0 0 1) case, the coiled conformation indicates the polymer exhibits an unfavourable interaction with the calcite as the polymer attempts to reduce the contact surface area with the less stable calcite structure.

The adsorption energy of polyacrylamide-based polymers on calcite can be attributed to three main contributions: (i) electrostatic interaction between the polymer O lone pairs of electrons and calcite Ca atom; (ii) electrostatic interaction between the polymer H atom and calcite O atom, or hydrogen bonding if the H atom is attached to the polymer highly electronegative atom (N and O); and (iii) dispersion interactions between all atoms of the polymer and the calcite surface. For the case of rigid calcite (0 0 1), the main functional group of PAM that contributes to the adsorption is the carbonyl group from the amide functional group (-C**O**NH_2_), with the O atom attracted to the Ca atom. We note that the surface consists purely of Ca^2+^. If the polymer is placed near the CO_3_^2−^ layer, it will be the H atom bonded with the N atom (-CON**H_2_**) attracted to the CO_3_^2−^. This suggests that the adsorption mechanism of the polymers to the calcite is dominated by the electrostatic interaction between the oppositely charged atoms. For the flexible and rigid calcite (1 0 4), since the surface layer of calcite (1 0 4) consists of both Ca^2+^ and CO_3_^2−^, the H atom and O atom from the amide functional group are attracted to the CO_3_^2−^ and Ca^2+^, respectively on the surface. A similar adsorption mechanism is also observed for neutral and ionised HPAM cases, in addition to the dominating carboxyl group (-CO**O**H) or carboxylate group (-CO**O**^−^) from acrylate copolymer contributing to the adsorption. 

To quantify the polymer adsorption behaviour on both calcite crystal planes, the interaction energy between them was assessed using LAMMPS compute *group/group* command, which is closely related to Equation (1)

(1) $$E_{interaction}=\frac{E_{polymer+calcite}-(E_{polymer}+E_{calcite})}{N}$$

where $E_{interaction}$ is the interaction energy between polymer-calcite adsorbed system, $E_{polymer+calcite}$ is the total energy of the polymer-calcite adsorbed system after equilibration, $E_{polymer}$ and $E_{calcite}$ is the total energy of the isolated polymer and calcite system, respectively, and N is the total number of polymer molecules. 

**Figure 3:**
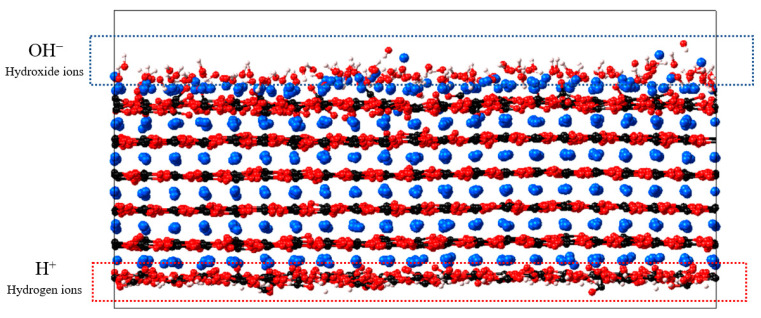
Equilibration of calcite (0 0 1) with hydroxide ions at the upper surface layer and hydrogen ions at the lower surface layer. The system is at a lower free energy state in comparison to the unmodified case.

For the flexible calcite (0 0 1) case, the polymer interaction energy measured is inconsistent among different realisations due to the unstable calcite structure. Therefore, a modified structure is proposed to stabilise the upper layer with OH^−^ ions (surface layer with Ca^2+^) and the lower layer with H^+^ ions (surface layer with CO_3_^2−^). As shown in Figure 3, after this modification, the calcite (0 0 1) is in a more stable state, and the interaction energy measured is reproducible. No modification is made to calcite (1 0 4).

**Figure 4:**
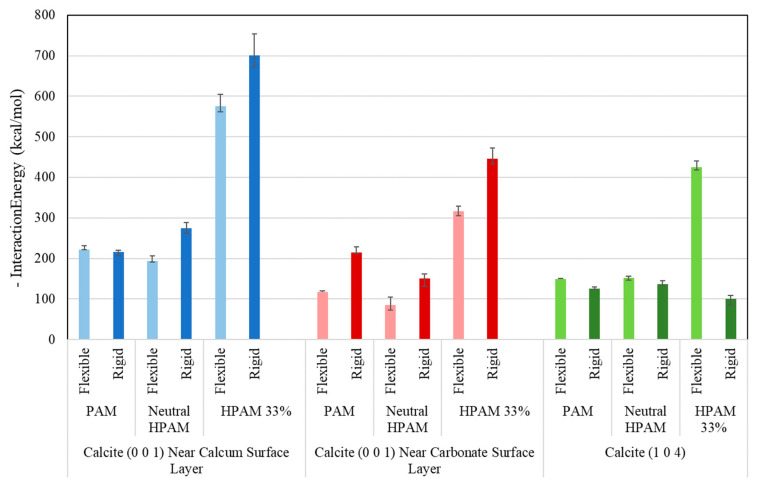
Interaction energy of calcite with polymers for different cases.

The interaction energy of different scenarios of calcite (0 0 1) and calcite (1 0 4) are depicted in Figure 4. Note that the negative interaction energy is plotted as an absolute value for better visualisation. Comparing the flexible and rigid cases, it can be observed that for the calcite (0 0 1), the interaction energy of all polymers in the rigid surfaces is typically higher than the flexible cases for the adsorption on both surface layers. We presume this may be due to the vibrational energy from the flexible calcite structure affecting the interaction with the polymers, resulting in reduced interaction energy compared to rigid cases. However, for calcite (1 0 4), the interaction energy of rigid and flexible cases is approximately equal, with the flexible cases slightly higher than the rigid cases. The only exception is the HPAM 33% in the flexible case, which has significantly higher interaction energy. This may be attributed to the acrylate monomer in the ionised form exhibiting stronger electrostatic interactions with the calcite surface, even though this effect is not captured in the rigid case.

When comparing the adsorption performance of different polymers, the HPAM 33%, an anionic polymer, generally performs better than neutral polymer (PAM and neutral HPAM) due to the presence of ionised acrylate monomer. Regarding crystal plane comparison, the polymer interaction energy for the calcite (0 0 1) case near the Ca^2+^ layer is typically higher compared to the adsorption near the CO_3_^2−^ layer and calcite (1 0 4) cases. As the polymer functional group attracted to the Ca^2+^ layer consists of the oxygen atom from the carbonyl group, this may explain why the interaction energy near the Ca^2+^ layer is higher due to the strong interaction with Ca^2+^ ions. This becomes more obvious in the case of HPAM 33% near the Ca^2+^ layer, as the acrylate monomer is deprotonated and ionised, more ionised oxygen atoms from the carbonyl group are available to interact with the Ca^2+^ layer, hence resulting in the highest interaction energy. This is consistent with the experimental and molecular studies reported by Legens et al. [35], which showed that surface calcium is the most probable adsorption site in these cases. 

**Figure 5:**
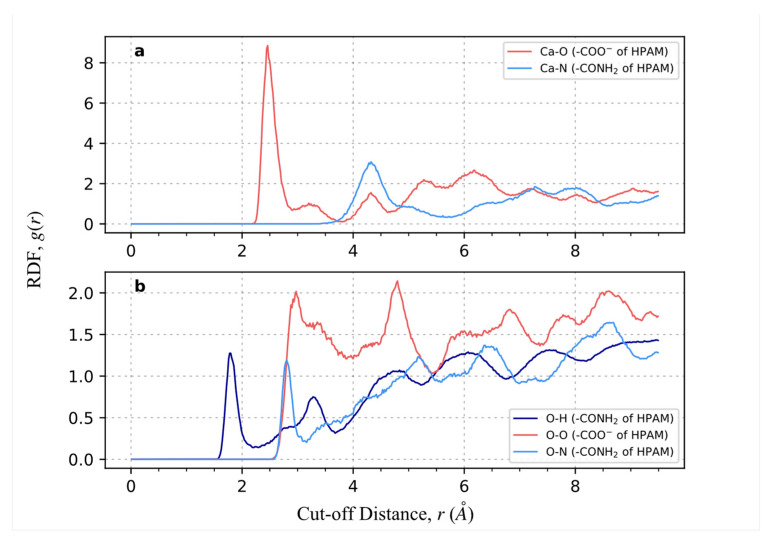
The radial distribution function of HPAM is 33% adsorption on calcite (1 0 4) surface. (**a**) Calcite Ca atom with electronegative O, N atoms of polymer. Red line refers to the O atom in the ionised acrylate monomer, and light blue line refers to the N atom in the acrylamide monomer (**b**) Calcite O atom with partially positive H atom and electronegative O, N atoms polymer. Red line refers to the O atom in the ionised acrylate monomer, light blue line refers to the N atom in the acrylamide monomer, and dark blue line refers to the H atom in the acrylamide monomer.

To support the above claims, the radial distribution function (RDF), also known as the pair correlation function, *g(r)*, is computed for HPAM 33% adsorption on calcite (1 0 4) surface, as it consists of both alternating CO_3_^2−^ and Ca^2+^ and provides fair comparisons to determine the dominant atom pairs contributing to the main adsorption mechanism. HPAM 33% consists of two electronegative atoms: the O atom in ionised acrylate monomer and the N atom in acrylamide monomer capable of exhibiting strong electrostatic interaction with calcite Ca atom. At the same time, hydrogen bonding can be achieved between the calcite O atom and the partially positive H atom attached to the N atom in the acrylamide monomer. As depicted in Figure 5, the peak in the curve of the *g(r)* Ca-O is higher than the *g(r)* of Ca-N, followed by the *g(r)* of O-H, indicating the probability of electrostatic interaction between the Ca-O atom pair is the highest. For the distance where the peak occurs, it can also be observed that the *g(r)* Ca-O appears around 2.5 Å at a significantly closer distance than the *g(r)* Ca-N, while the *g(r)* O-H appears at a shorter distance of 1.75 Å due to H atom with a smaller radius. The *g(r)* O-O and *g(r)* O-N are also included to show the electrostatic repulsion between the same charge atoms pair. Overall, the analysis of the RDF supports the conclusion that the electrostatic attraction between the O atom and Ca atom dominates the polymer adsorption on calcite. From the interaction energy decomposition analysis (see Table S7 in Supplementary Information), the Ca-O atom pair is shown to exhibit the largest contribution to the coulombic interaction, whereas the vdW contributions are an order of magnitude lower as a result of the ionic crystal structure. 

To conclude, upon evaluating both the polymer adsorption conformations and interaction energy analysis on calcite crystal planes, it is seen that the flexible calcite (1 0 4) is the most appropriate surrogate of carbonate reservoirs for further studies. Even though the interaction energies near the calcium (0 0 1) surface for all polymers are generally higher, the surface is unrepresentative as it is layered with only Ca^2+^ ions, contributing to the abnormally high value. Meanwhile, the calcite (1 0 4) structure has CO_3_^2−^ and Ca^2+^ ions in the same layer and is unaffected by the different surface layers. It is proven more thermodynamically stable without artificial inclusions of other ions to equilibrate and stabilise the structure. Moreover, from the interaction energy analysis, the flexible and rigid structure of calcite (1 0 4) also does not deviate from each other significantly, while the flexible calcite (1 0 4) shows a more realistic representation of interaction energy with anionic polymer and consideration of calcite vibrational energy. This is supported by literature claims that the calcite (1 0 4) is the most thermodynamically stable structure and has the lowest surface energy compared to other crystal planes [3,21,36,37]. As such, the flexible calcite (1 0 4) will be used in the remainder of the studies reported herein.

### 2.2. Umbrella Sampling Adsorption Free Energy Analysis

With the selection of an appropriate calcite crystal plane, the main research interest is to develop a systematic simulation protocol to screen the polymer adsorption performance. Adsorption-free energy analysis with enhanced sampling has been applied to aid the analysis. This is useful in solvent systems with a free energy barrier, where the biased potential can help sample the system’s conformational spaces more efficiently. In our study, the polymer adsorption performance will be screened in vacuo first, and the same simulation protocol will be applied in the presence of the solvent phase.

From our preliminary studies, the US enhanced sampling parameters, such as force constant, windows spacing interval and total windows sampling distance, are refined to ensure well overlapping between the window stages. This can be observed from the trajectory probability density distribution in the histogram, where an example of a histogram for the adsorption of PAM on calcite (1 0 4) is depicted in Figure S9 in Supplementary Information.

Note that for the anionic polymers of HPAM 33% and SPAM 33%, the simulation is performed without the placement of counterions. Even though it is common practice to ensure the electroneutrality of the simulation environment, for the free energy analysis in vacuum conditions, the placement of counterions will disrupt the free energy due to the ion shielding effects and result in severe sampling problems. A similar finding has been reported in the analysis of hydration-free energy of neutral and charged solute, where the simulation environment without the counterions can reach sampling convergence and minimal disruption effect in comparison to the placement of counterions [38]. 

**Figure 6:**
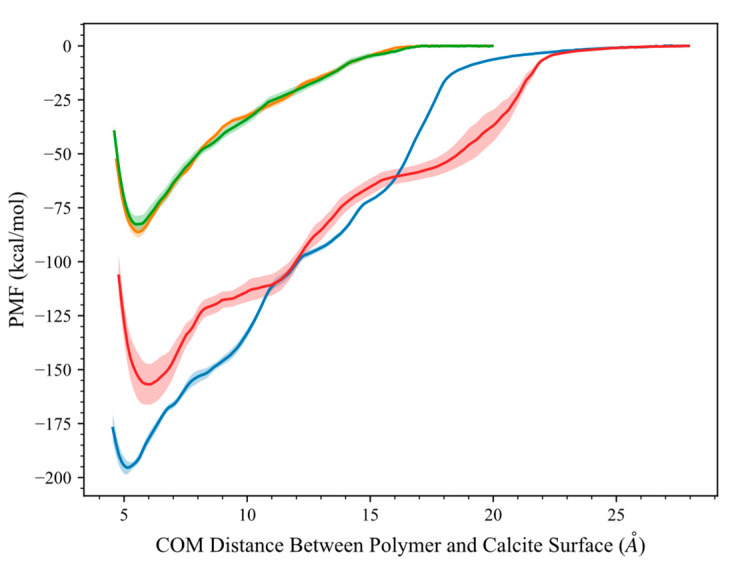
Adsorption free energy profile of polymers with calcite at 298 K. Calcite (1 0 4) plane is located at the origin. Curves correspond to the average of 5 different realisations, with the shaded area as standard error from the average results. Colour indications—Green: PAM, Orange: Neutral HPAM, Blue: HPAM 33%, Red: SPAM 33%.

A comparison of the free energy profile of the adsorption of different polymers on calcite (1 0 4) is included in Figure 6. The minimum in the free energy indicates the most stable state, and higher values reflect the adsorption preference of the polymer on calcite. For the neutral versions of PAM and HPAM, their free energy values are essentially the same and the least negative. Meanwhile, HPAM 33% has lower free energy compared to other polymers, presumably due to the presence of deprotonated carboxyl groups from the acrylate copolymer. This indicates the importance of the hydrolysis process to deprotonate the polymer and convert it into an anionic polymer. In its hydrolysed form, HPAM has a strong electrostatic interaction with the calcite structure, improving the chemical adsorption performance.

The comparison of the results between HPAM 33% and SPAM 33% agree with the trends obtained from experimental results as tabulated in Table 1, where there is a higher equilibrium adsorbed amount of HPAM polymer on the calcium carbonate sample compared to SPAM polymer. The AFM results confirm that the adhesion force of HPAM 33% on calcite surface is stronger than SPAM 33%.

The stronger adsorption of HPAM 33% over SPAM 33% can be explained in terms of classical thermodynamics. If the pressure-volume contribution (PV) can be ignored, the Helmholtz free energy computed in the *NVT* ensemble can be assumed numerically equal to the Gibbs free energy, which can be broken down in terms of both enthalpic ($∆H_{ads}$) and entropic contributions ($-T∆S_{ads}$) [8,39]. Some insights on the extent of the enthalpic and entropic contribution to the adsorption-free energy of polymers have been reported in the literature. For example, de Angelis et al. [10] studied the adsorption-free energy profile of pulling a surfactant slowly to a bare or surfactant-coated nanoparticle (NP) surface. Even though the dispersion interaction and adsorption enthalpy between the surfactant and surfactant-coated NP increase, the competing entropic contribution from the steric effects in the surfactant-coated NP case reduces the overall free energy profile compared to bare NP. Similarly, Willemsem et al. [8] assessed the adsorption of phthalate esters on clay surfaces, where they detail the adsorption on a clay interlayer region of a bulky phthalate ester provides unfavourable free energy due to steric hindrance and potential entropic penalty.

We propose that a similar phenomenon can be observed in our study. HPAM 33% and SPAM 33% can be considered as large macromolecules, and even though the adsorption enthalpy has a significant contribution and entropy has a minor contribution [40], the entropic loss will contribute to the repulsive interaction. During the adsorption process, the entropy loss due to the decreasing conformational freedom results in a positive entropic contribution and reduces the overall free energy magnitude. For SPAM 33%, the steric hindrance effect from its bulkier functional group is more significant, contributing to the higher entropy penalty [8,10]. Therefore, its adsorption-free energy is less than HPAM’s 33%, which has been proven and reflected in the experimental results.

#### Evaluation of HPAM 33% Adsorption on Calcite in the Presence of Solvents

**Figure 7:**
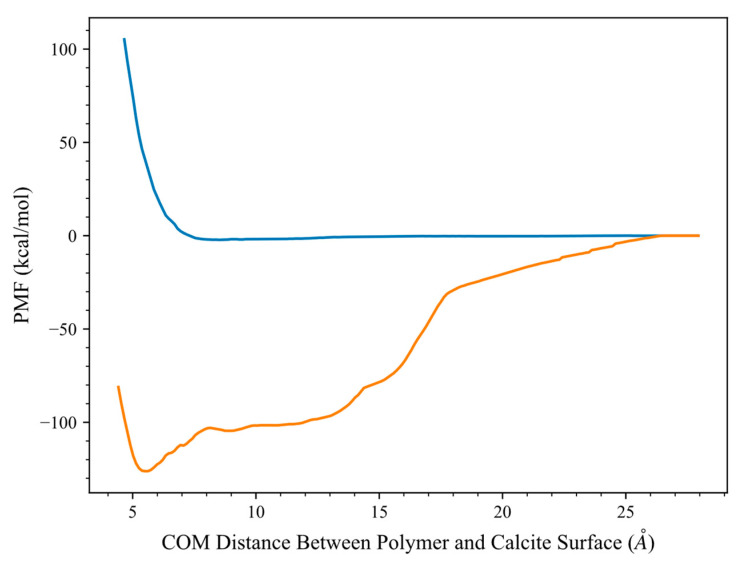
Adsorption free energy profile of polymer HPAM 33% with calcite at 298 K under the presence of different solvents. The calcite (1 0 4) plane is located at the origin. Colour indications—Blue: solvent water, Orange: solvent dodecane.

Further adsorption-free energy analysis in the presence of the solvent is conducted for HPAM 33%. The main interest is to see the effect of the polarities of different solvents, taking as a benchmark the free energy in vacuum conditions. As shown in Table 2 and Figure 7, the adsorption-free energy magnitude of HPAM33% polymer decreases drastically in water, indicating the significance of the water shielding effect on polymer adsorption. Water as a polar solvent can form strong hydrogen bonds with the polymer; thus, when water molecules surround the polymer, the polymer polarised functional group will develop a favourable interaction with the water molecules relative to the calcite structure, which hinders the polymer adsorption. This results in the preference of the polymer to remain in the water phase rather than in the adsorbed state on calcite. A similar solvent effect is also reported in the literature, where there is a suggestion that water induces a repulsive contribution to the PMF between the bare graphitic nanoparticles interaction [41] and weakens the PAM adsorption on the polar cellulose nanocrystal [24]. A study related to the oil recovery mechanism also indicated that the adsorption-free energy between the oil molecules and the hydrophilic surfaces of kerogen and calcite is significantly weakened under the presence of water, which facilitates the oil recovery process [11].

On the other hand, in the presence of dodecane, the adsorption-free energy of the HPAM 33% is closer to the vacuum condition case. The lower free energy computed is presumed to be due to the shielding interaction of the dodecane molecules compared to the in vacuo case. As dodecane is a non-polar molecule, the polymer has unfavourable interactions with the organic phase and prefers to adsorb on the calcite surface rather than staying in the oil phase, resulting in adsorption-free energy on calcite higher than water case. Overall, the free energy analysis in the presence of solvents provides insight into how the solvent polarity affects the polymer adsorption performance. To screen polymer adsorption performance, these effects need to be considered, especially for the application of the polymer treatment in a reservoir environment, where the reservoir composition and solvent polarity will impact the adsorption performance.

We highlight that in this research, we focused on the variation of PAM polymer candidates, consisting of a relatively short oligomeric chain. Experimental evidence [30,31] and simulation results [8,42] demonstrate that the polymer conformations can be influenced by the chain length and other parameters, which may affect the interaction behaviour and the adsorption-free energy on the calcite surface. For example, a polymer with a shorter chain length and strong charge density adopts extended conformation and develops a favourable adsorption towards the surface. However, with increasing chain length under the same environment, the polymer may adopt a coiled conformation due to increasing compacity and will find it more difficult to adsorb to the surface completely. Future research work in our group will be focused on the polymer adsorption performance under different parameters to gain insights into the adsorption mechanism.

## 3. Materials and Methods

### 3.1. General Simulation Details

Classical atomistic molecular dynamics (MD) simulation is employed using Material Exploration and Design Analysis (Medea) simulation software version 3.5 [43] integrated with the LAMMPS module [44] with a built-in visualisation interface. The intra-atomic and inter-atomic potentials are described by the all-atom Polymer Consistent Forcefield-enhanced version (PCFF+). Its structure is based on the COMPASS force field [45] and its derivation from the original PCFF, which extensively parameterised and validated the properties of polymers and other condensed-phase materials [46]. The bonded terms consist of bond stretching, angle bending, and torsional angle rotation terms. The non-bonded terms consist of a 9-6 Mie potential plus fixed point Coulombic electrostatic interactions [45]. A table of parameters, Table S1, is included in the Supplementary Information.

Periodic boundary conditions are applied in all Cartesian directions. The non-bonded interactions and real-space contribution of the Coulombic energy are computed with a cut-off distance of 9.5 Å, with the reciprocal-space contribution computed with the particle-particle-particle Mesh (PPPM) method [47]. The velocity Verlet algorithm is used with a timestep of 1 fs. The system energy is minimised using the conjugate gradient method, and initial random velocity is assigned to all the atoms at a specified temperature of equilibration and production run. Whenever an isobaric-isothermal ensemble (NPT) or a constant density-isothermal ensemble (NVT) is performed, the system is controlled using the Nose-Hoover thermostat and barostat with the correction terms of Martyna, Tuckerman, and Klein [44,48] included in the equations of motion.

An essential aspect of any simulation study is the validation of the forcefields for the particular task at hand. While the accuracy of molecular simulations is frequently taken for granted, it has been shown that this premise can be significantly flawed [49]. To ensure PCFF+ is suitable for modelling the polymer adsorption performance, different validation works have been performed, such as validation of the calcite structural properties and solid-fluid interaction (enthalpy of immersion of different calcite-solvent systems and adsorption energy evaluation of different functional groups of organic molecules on calcite). The methodologies and results are included in Supplementary Information, where the results have been supported with literature, indicating the applicability of the chosen forcefield for our study.

### 3.2. Polymer Models 

Polyacrylamide-based polymers studied include polyacrylamide (PAM), hydrolysed polyacrylamide in neutral form (neutral HPAM), hydrolysed polyacrylamide with 33% charge density (HPAM 33%), and sulfonated polyacrylamide with 33% charge density (SPAM 33%). The oligomer compositions and molecular weight are included in Table 3. To model the HPAM 33% repeat unit, each deprotonated acrylate monomer was paired with two acrylamide monomers. This arrangement resulted in a polymer with 33% negative charge density for a given repeat unit and a 100% degree of ionisation for the whole polymer chain. To investigate the effect of ionisation, the same structure of HPAM 33% but devoid of deprotonation was created as neutral HPAM.

### 3.3. Calcite Model 

A unit cell of the rhombohedral calcite crystal structure of space group *R*$\bar{3}$c is employed based on the diffraction data of Antao and Haslan [50]. The unit cell has a dimension of *a* = *b* = 4.980 Å, *c* = 17.192 Å, and a plane angle of α = β = 90°, γ = 120°. Periodic boundary conditions are applied in the surface (*x*,*y*) directions. When included in a larger cell (e.g., when considering solvents, polymers, etc.), the solid forms part of a much larger cell with periodic boundary conditions in the *z* direction. 

#### Crystal Plane and Rigidity of the Calcite Model

Creating the most suitable calcite slab model for polymer adsorption performance is essential to ensure the realistic presentation of the adsorption properties. The unit cell described above is further cleaved into crystal planes (0 0 1) and (1 0 4) with six layers of thickness. Calcite (0 0 1) structure has a dimension of *a* = 49.0 Å, *b* = 51.0 Å and *c* = 18.84 Å, while calcite (1 0 4) has a dimension of *a* = 48.86 Å, *b* = 49.76 Å and *c* = 17.84 Å.

A single molecule of each polymer is placed close to the calcite (0 0 1) and (1 0 4) surfaces in a vacuum cell to assess the interaction behaviour. Note that SPAM 33% polymer is not involved in this section, as the focus is to evaluate the suitability of calcite crystal plane structure. The calcite (0 0 1) structure is asymmetric, having Ca^2+^ ions on one surface layer and CO_3_^2−^ ions in the opposite layer, where the surface layer of different charges may affect the polymer adsorption. Secondly, most of the studies in the literature considered calcite in a rigid structure and neglected the calcite vibrational energy. Such an approach might not be suitable for solids production control cases as the calcite vibrational energy may affect the chemical adsorption performance. Moreover, a preliminary study shows that when allowing the calcite (0 0 1) structure to equilibrate freely, it is highly unstable, and the atoms vibrate vigorously in their position (see Figure 1c), while calcite (1 0 4) vibrates stably and do not have this problem.

Based on these considerations, the simulation is performed in both cases of either allowing the calcite structure to move freely (flexible) or to freeze its movement (rigid). To stabilise the atomic movement of calcite (0 0 1), it is equilibrated with a layer of 120 molecules of hydrogen ions at the bottom surface (with 120 molecules of CO_3_^2−^ ions) and a layer of 120 molecules of hydroxide ions at the top surface (with 120 molecules of Ca^2+^ ions). For calcite (0 0 1), the polymers are placed near the top CO_3_^2−^ surface layer or the bottom CO_3_^2−^ layer to examine the surface layer effect. This results in four different cases: (1) rigid calcite case and near-CO_3_^2−^ layer; (2) rigid calcite case and near-Ca^2+^ layer; (3) flexible calcite case and near-CO_3_^2−^ layer; and (4) flexible calcite case and near-Ca^2+^ layer. For calcite (1 0 4), as the structure consists of both Ca^2+^ and CO_3_^2−^ ions in the same layers, and the structure can equilibrate stably, the polymer is placed directly near the top calcite surface, and the rigid and flexible cases are evaluated.

The polymer is placed into the simulation system with a vacuum layer thickness of 50.0 Å to prevent the polymer from interacting with the opposing surface layer. The PCFF+ force field is employed for all atom types. The simulation is performed under the NVT ensemble at 298 K for 500 ps. The simulation is repeated four times for each case, with different initial random positions on the surface plane. The equilibrated conformation is evaluated, while the average interaction energy between the polymer and calcite is analysed using the LAMMPS *compute group/group* command. The most suitable crystal plane is determined, and the radial distribution function (RDF) analysis is conducted to determine the responsible functional group for the polymer adsorption.

### 3.4. Umbrella Sampling Adsorption Free Energy Analysis

To assess the polymer adsorption performance, the adsorption-free energy is analysed using an enhanced sampling method, Umbrella Sampling (US). The free energy analysis is conducted on flexible calcite (1 0 4) case in vacuum conditions with the same simulation system in Section 3.3. This is performed using the COLVARS module integrated into LAMMPS. The reaction coordinate is defined as the distance in *z*-direction between the centre of mass (COM) of the first two layers of calcite (reference group) and the COM of the polymer molecule (pulling group). For a neutral polymer, a total of 81 window stages with a window spacing interval of 0.2 Å is used, resulting in a total sampling distance of 16 Å (20 Å in the bulk region to 4 Å from calcite surface COM). For the anionic polymers, HPAM 33% and SPAM 33%, as the polymers have stronger interaction with the calcite, the total sampling distance is increased to 24 Å (28 Å to 4 Å from calcite surface COM) with 121 window stages. The applied biased potential force constant is set as 100 kcal/mol.Å^2^. A fast series US simulation with a sampling time of 10 ps is performed at each window to prepare the initial conformation of the polymer. Further, a more refined US simulation is performed with a parallel simulation of all windows conducted for 6 ns with the trajectory frequency sampled every 100 fs. For each window case, the polymers can rotate and equilibrate freely across the *xy* plane in the specified restraint centre. The first 2 ns of simulation is discarded to ensure equilibration. All simulations are performed under the canonical NVT ensemble at 298 K. The remaining trajectory samples for all windows are post-processed with the WHAM free energy estimator tool to produce the global free energy profile, with the number of bins set as 200. For each polymer case, five different realisations are computed to obtain the average free energy profile.

#### Free Energy Analysis in the Presence of Solvents

To investigate the effect of solvent polarity on the free energy, a US simulation is conducted for the HPAM 33% case in the presence of a water phase and an oil phase represented by pure n-dodecane. In a separate system, 9000 water molecules and 750 dodecane molecules are stacked with the calcite structure. To eliminate the vacuum region between the solvent and calcite, the system is equilibrated under the constant temperature and constant pressure in z direction (NPzzAT) ensemble for 1 ns at 298 K and 1 atm followed by simulations in the NVT ensemble for 4 ns. The NPzzAT ensemble allows the system to be taken to the correct pressure level without disrupting the crystal structure. In this ensemble, the barostat operates by changing the volume of the simulation box in a direction (*z*), keeping the other two dimensions constant [39]. The HPAM 33% polymer is then placed into the system to perform the free energy adsorption analysis with the same US simulation protocol for the HPAM 33% case as in the vacuum condition.

### 3.5. Experimental Adsorption Studies and AFM Analysis

#### 3.5.1. Material

The anionic polyacrylamide (PAM) used in this work (F3330S: 30% hydrolysed, 11–13 MDa; AN125: 25% sulfonated, 8MDa) is provided by SNF Floerger (Wakefield, UK). Calcium carbonate powder (CaCO_3_, ≥99%) and sodium chloride (NaCl, 99.5%) are purchased from VWR Chemicals Ltd. (Lutterworth, UK), while Iceland Spar calcite crystals used for Atomic Force Microscopy (AFM) work are purchased from Manchester Minerals (Shrewsbury, UK). Polymer mother stock solutions of 15,000 ppm were prepared according to API-RP-63, in which diluted stock solutions would be prepared by dilution using DI water for the adsorption study and with NaCl solution for the AFM study. The solvent used in this work is deionised water (DI, 18 MΩ Ohm), and all experiments are conducted in room conditions.

#### 3.5.2. Adsorption Study

Adsorption of PAM onto CaCO_3_ is studied via UV-Vis spectrometry. 1 g of CaCO_3_ is added into 25 mL of diluted polymer solutions and stirred for more than 18 h, the reported minimum time required for equilibrium adsorption of HPAM onto CaCO_3_ [30]. After the adsorption, the mixtures are centrifuged at 8500 RPM for 40 min, after which the supernatant is analysed using the Shimadzu UV2600 spectrophotometer to determine the amount of HPAM adsorbed (further details are given in ref. [29]). The amount of PAM adsorbed per unit surface area CaCO_3_, *Q_e_* (mg/m^2^) is then calculated as the difference between the initial and equilibrium concentration of the supernatant.

#### 3.5.3. Atomic Force Microscopy (AFM) Analysis

Atomic Force Microscopy (AFM) study is conducted using a JPK NanoWizard 4 atomic force microscope with NanoWorld^®^ Pyrex-Nitride Probe—Triangular AFM Cantilevers (PNPTR) (nominal force constant: 0.32 N/m, cantilever length: 100 µm, tip height: 3.5 µm, tip radius: <10 nm). To ensure the polymer interaction is always with CaCO_3_, CaCO_3_ particles are attached to the AFM tip using a micromanipulator (Melles Griot, Rochester, NY, USA) under a microscope. The calcite crystals were scanned primarily via Force Mapping (FM) mode (setpoint: 0.5 nN; vertical length: 300–500 nm; contact time: 2 s; pixel: 16 × 16) to study the surface interactions regarding force spectroscopy profiles. A minimum of four scans are conducted, with the entire experiment repeated twice using a freshly cleaved calcite surface and a new cantilever. The interaction energy obtained from the AFM measurements can be directly compared to the MD simulation results, with the proviso that the molecular weights of the two systems are very different.

## 4. Conclusions

Polyacrylamide-based polymers have been modelled using classical atomistic MD simulations. The forcefield employed to model the polymer adsorption performance on calcite is successfully validated against experimental data and is favourably compared to existing simulation literature. To ensure the most realistic representation of a reservoir rock, calcite crystal planes (0 0 1) and (1 0 4) are evaluated and compared in terms of polymer adsorption conformation and interaction energies. Flexible calcite (1 0 4) is the most suitable surrogate due to its structural stability and polymer adsorption characteristics. Adsorption-free energy analysis is computed with umbrella sampling simulations to screen the polymer adsorption performance. The adsorption free energy profile indicates HPAM 33% has the most negative free energy attributed to the presence of deprotonated acrylate copolymer, capable of developing strong electrostatic interactions with calcite structure. Compared to other polymers, the improved chemical adsorption performance is also consistent with the indication from experimental adsorption and AFM results. While SPAM 33% also has favourable adsorption to calcite, its bulkier functional groups limit its adsorption effectiveness compared to HPAM 33%. Solvent polarity will reduce the adsorption-free energy due to the solvent shielding interactions, with a significant reduction seen when solvated in water and a relatively lesser reduction when in dodecane. Overall, this study provides insights into the polyacrylamide polymer adsorption mechanism on calcite and suggests avenues to optimise the design of additives for reservoir rock strengthening. 

## Figures and Tables

**Table 1:** Comparison of polymer adsorption-free energy with the indications from the experimental study.

Polymer Type	MD Simulation	Experiment	
	Adsorption Free Energy (kcal/mol)	Equilibrium Adsorbed Amount (mg/m^2^)	AFM Interaction Energy (aJ)
HPAM	197	0.25	34.903
SPAM	160	0.14	9.413

**Table 2:** Adsorption-free energy of HPAM 33% in the presence of solvents.

	Vacuum	Water	Dodecane
Negative adsorption-free energy (kcal/mol)	197	2.25	126.2

**Table 3:** Polymer compositions and molecular weight. x and y refer to the ratio of copolymers in a repeat unit.

Polymer	Repeat Unit Composition	Copolymer Ratio/Total Repeat Units for a Polymer Chain	Molecular Weight (Da)
PAM	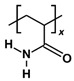	*x* = 3, repeat units = 3	641.725
HPAM 33%	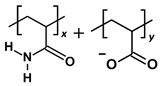	*x:y =* 2:1, repeat units = 3	641.656
Neutral HPAM	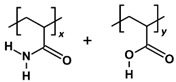	*x:y =* 2:1, repeat units = 3	644.680
SPAM 33%	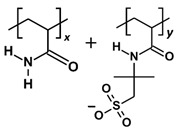	*x:y =* 2:1, repeat units = 3	1116.186

## Data Availability

Data available from the authors upon reasonable request.

## Supplementary Materials

The following supporting information can be downloaded at: doi.org/10.6084/m9.figshare.23587482 (accessed on 27 June 2023). Supplementary Information is provided with this manuscript, including forcefield parameters, validation of solid-fluid interactions and example input files. Refs. [51,52,53,54,55,56,57,58,59,60] are cited in Supplementary Materials.

## Sample Availability

Samples of the anionic polyacrylamide and calcium carbonate powder are available from J.H.L.
